# Green-synthesized tellurium nanoparticles as a multifunctional leather finishing agent: antimicrobial and mechanical enhancement

**DOI:** 10.1038/s41598-026-57078-0

**Published:** 2026-06-16

**Authors:** Shereen A. Abdeldayem, Radwan Mohamed Ali, Khaled Sayed-Ahmed

**Affiliations:** 1 Department of Textile Printing, Dyeing & Finishing, Faculty of Applied Arts, Damietta University, Damietta, Egypt; 2 Biochemistry Department, Faculty of Agriculture, Al Azhar University, Cairo, Egypt; 3 Department of Agricultural Biotechnology, The Centre for Excellence in Research of Advanced Agricultural Sciences, Faculty of Agriculture, Damietta University, New Damietta, Egypt; 4 Department of Chemistry, Faculty of Science, New Mansoura University, New Mansoura, Egypt

**Keywords:** Leather, Green synthesized, Redox reaction, Plant extract, Antibacterial and antifungal activities, Biotechnology, Chemistry, Materials science, Microbiology, Nanoscience and technology

## Abstract

Leather is highly susceptible to microbial colonization due to its moisture retention and nutrient-rich structure, which promotes bacterial and fungal growth and compromises hygiene, durability, and user comfort in applications such as footwear and upholstery. In this work, tellurium nanoparticles (TeNPs) were green synthesized through redox reaction using plant extract and structurally characterized by X-ray diffraction (XRD) and transmission electron microscopy (TEM) to confirm crystalline phase, particle size, and morphology. The TeNPs were then applied to leather substrates to generate antimicrobial leathers. The distribution and surface morphology of TeNPs on leather were examined using scanning electron microscopy (SEM) and energy-dispersive X-ray spectroscopy (EDX) to verify nanoparticle anchoring and elemental composition. Mechanical performance was assessed through tensile strength and elongation testing to evaluate the influence of TeNP incorporation on the integrity of the leather matrix. Antibacterial and antifungal activities of TeNP-treated leather were quantified against representative Gram-positive and Gram-negative bacteria and Aspergillus fungal. The antimicrobial efficacy of TeNPs is attributed to ROS generation, disruption of microbial cell membranes, inhibition of thiol-containing respiratory enzymes, and Te redox cycling-mediated oxidative stress, which together achieve broad-spectrum inactivation of bacterial and fungal pathogens. The TeNP-functionalized leather exhibited pronounced antibacterial and antifungal activities while enhancing mechanical performance, indicating its potential as a high-value, hygienic leather material for advanced footwear and other consumer applications.

**Supplementary Information:**

The online version contains supplementary material available at 10.1038/s41598-026-57078-0.

## Introduction

The growing demand for high-value leather in medical, protective, automotive, footwear, and luxury sectors is driving the development of multifunctional, bio-safe surfaces with long-lasting antimicrobial protection. Conventional tanning and finishing rely largely on organic biocides or metallic salts, which can lose activity over time and raise environmental and toxicological concerns. In parallel, the worldwide rise of multidrug-resistant bacteria colonizing animal-derived materials, especially in biomedical contexts, underscores the need for durable and safe antimicrobial strategies closely aligned with industrial practice and market demands. It should be noted that the term ‘green’ in this study refers exclusively to the plant extract-mediated synthesis of TeNPs and their application as a post-tanning finishing agent. The conventional tanning workflow employed herein is not claimed to be environmentally benign. The TeNP finishing step is designed as a modular, add-on treatment applicable to any conventionally or alternatively tanned leather substrate, thereby enabling functional upgrading of existing leather products without modifying the upstream processing chain.

Nanotechnology-based finishes using metal and metal-oxide nanoparticles already offer promising solutions for antimicrobial and functional leather. Ag-, TiO₂-, CuO-, MgO-, and SiO₂-containing nanocomposites, xerogels, and hybrid systems have been applied as eco-friendly coatings to leather, improving antibacterial and antifungal performance, UV resistance, hydrophilicity/hydrophobicity balance, abrasion resistance, and mechanical durability, often with low cytotoxicity and compatibility with standard finishing lines^1–9^. These multifunctional coatings are attractive for cost-effective and scalable production of high-value hygienic leathers and are increasingly considered for industrial implementation in footwear, automotive interiors, and medical leathers^2,10,3–5,11^.

Significant recent advances have been made in sustainable and cleaner leather processing, including the development of chrome-free tanning systems, bio-based crosslinkers, and waterborne finishing formulations that substantially reduce the environmental footprint of leather production [see e.g.,^12^;^13^;^14^;^15^. These advances underscore the growing imperative to develop finishing technologies that not only deliver functional performance but are also compatible with cleaner production frameworks. In this context, plant-mediated green synthesis of nanoparticles—leveraging phytochemical reductants and stabilizers under mild, solvent-free aqueous conditions—represents a synthesis paradigm fully aligned with green chemistry principles, offering a viable route to functional nano-finishes without the environmental burden of conventional chemical reduction methods.

Tellurium nanomaterials have recently emerged as potent broad-spectrum antimicrobials with activity against Gram-positive and Gram-negative, including multidrug-resistant strains, and with demonstrated ability to disrupt biofilms and promote infected wound healing in vivo^16–22^. Biocompatibility data for TeNPs remain concentration-dependent; cytotoxicity toward mammalian cells has been reported at high doses, while optimized formulations have shown acceptable safety profiles in specific in vitro models^22^. Accordingly, biocompatibility assessment of the TeNP–leather system developed here is designated as a critical objective for follow-up work prior to any commercial application. Yet, current Te-based systems are mainly designed for temporary biomedical applications (e.g., wound dressings, implant-related coatings), and no reports have described Te nanostructures engineered as permanent leather finishes that tailor both the physicochemical and antimicrobial properties of leather substrates.

This work addresses that gap by developing a green, plant-based tellurium nano-finishing compatible with conventional tanning and finishing workflows, aiming to impart durable antibacterial and antifungal activity while enhancing leather’s surface and mechanical performance. By enabling scalable production of high-value hygienic leathers, this approach directly supports product quality, user safety, and economic competitiveness in consumer and technical leather markets. The global antimicrobial leather market is growing rapidly, driven by demand for hygienic footwear, automotive, and medical leather. TeNP finishing — using low-cost plant biomass under mild aqueous conditions compatible with existing tanning infrastructure — offers a cost-effective route to meet this demand.

## Materials and methods

### Materials

*Pluchea dioscoridis* leaves used in this study were harvested from the farm of agriculture faculty, Damietta university, Egypt. Leaves were collected according to institutional, national, and international guidelines and legislation. They were then washed using distilled water to remove impurities from the leaves surface, following by drying process at RT (25 ± 2 °C). All chemicals used in NPs synthesis or fabrication of TeNPs/leather were purchased from Sigma-Aldrich and used without any purification. In this study, cowhide was first processed into wet-blue leather using a conventional tanning recipe (Table 1). The workflow then comprised four main stages: (i) green synthesis of TeNPs using *Pluchea dioscoridis* leaf extract; (ii) decoration of tanned leather with TeNPs under varied temperature, time, and pH conditions; (iii) structural and surface characterization of TeNPs and TeNPs/leather by TEM, XRD, SEM, and EDX; and (iv) evaluation of mechanical properties and antibacterial/antifungal activities of untreated and TeNP-treated leather.

### Treatment of leather

Salted wet cowhide was processed into wet-blue leather through a conventional multi-stage tanning sequence comprising: pre-soaking and unhairing (lime/sodium sulfide, pH 12.5–13), deliming and bating (ammonium salts, Orpone protease, pH 8.5–9), pickling (formic acid/sulfuric acid, pH 3–3.5, 9 Bé), chrome-free vegetable tanning (Mimosa/Quebracho/sodium formate, pH 4–5), re-tanning (acrylic and phenolic syntans, Mimosa, Quebracho), fatliquoring (sulphited and sulphonated oils), and fixation (formic acid, pH 3.5). The complete tanning recipe is provided in Supplementary Table S1, following the protocol described by Ali et al.^23^.

### Green synthesis of TeNPs

TeNPs were prepared *via* a green synthesis method through redox reaction using plant extract (PE), as a reductant, and potassium tellurite (K₂TeO₃; MW = 253.8 g/mol), as a precursor. Aqueous plant extract was prepared by boiling 10 g of the dried *Pluchea dioscoridis* leaves into 400 mL of distilled water for 15 min. The boiling solution was then cooled at RT for about 10 min, followed by filtration through filter paper to remove the plant residue and obtain the filtrate. Additionally, 200 mL of potassium tellurite at a concentration of 0.01 M were mixed well with 200 mL of the prepared of aqueous plant extract and kept at RT for approx. 48 h to allow the reduction of tellurium ions to TeNPs. The change in color from yellowish green to dark black color indicated the complete formation of TeNPs. The synthesized TeNPs colloidal solution were further utilized to fabricate an antimicrobial leather^24^. The pH of the freshly prepared TeNPs colloidal solution was measured and adjusted to the desired values (pH 5, 7, or 9) before use in leather treatment experiments.

### Decoration of leather with TeNPs

Prior to treatment, the leather samples were scoured with a 20% acetone solution based on the weight of the leather at room temperature for 30 min with a liquor ratio of 1:50 to remove surface impurities. After scouring, the samples were thoroughly rinsed with distilled water before being immersed in TeNPs solution for further treatment.

In brief, the decoration of leather with TeNPs was conducted under a range of experimental conditions, including variable temperatures (at room temperature, 50 °C and 70 °C), different treatment durations (30, 60, and 90 min), and varying pH levels (5, 7, and 9), to evaluate the influence of these parameters on the functionalization process. Subsequently, the leather samples were thoroughly rinsed with distilled water and dried in an oven at 50 °C for 20 min. Finally, the dried samples were placed in a desiccator to ensure complete removal of residual moisture prior to further analysis.

### Transmission electron microscopy (TEM) and X-ray diffraction (XRD) Analysis

TeNPs size and morphology were examined using a transmission electron microscope (Talos L120C G2, Thermo Fisher Scientific) operated at 120 kV. A drop of the colloidal solution of TeNPs was loaded onto a 400-mesh copper grid coated with carbon film, followed by air-drying at RT (25 ± 2 °C). Additionally, the crystalline nature of the prepared TeNPs was determined using an X-ray diffractometer (Bruker D8 ADVANCE, Karlsruhe, Germany).

### Leather characterization

#### SEM and EDX analysis

The morphology of the untreated leather surfaces, and those treated with TeNPs were examined using a scanning electron microscope (JEOL JSM-6510LB, Tokyo, Japan) to confirm the sufficient coating of leather surfaces with the prepared TeNPs/leather. As for EDX, it used to identify and quantify the chemical makeup of TeNPs/leather.

### Physical properties of decorated leather with TeNPs

Tensile strength and elongation at break were measured once for both untreated and TeNPs-treated leather following ASTM D 4632; replicate mechanical tests and corresponding standard deviations were not obtained due to material and facility constraints.

#### Antibacterial and antifungal activities

To evaluate the antibacterial efficacy of both untreated and TeNP-treated leather, the zone of inhibition (ZOI) was measured in millimeters against four microbial strains: *Pseudomonas aeruginosa*,* Bacillus cereus*,* Aspergillus niger*, and *Aspergillus flavus.*

#### Statistical analysis

All quantitative data are expressed as mean ± standard deviation (SD). Differences among treatments were evaluated by one-way analysis of variance (ANOVA)^25^, and mean separation was performed using Duncan’s new multiple range test at *P* = 0.05^26^, using the Costat system for Windows, Version 6.311 (CoHort software, Monterey, CA, USA).

## Results and discussion

### Transmission electron microscopy (TEM) analysis

**Fig. 1:**
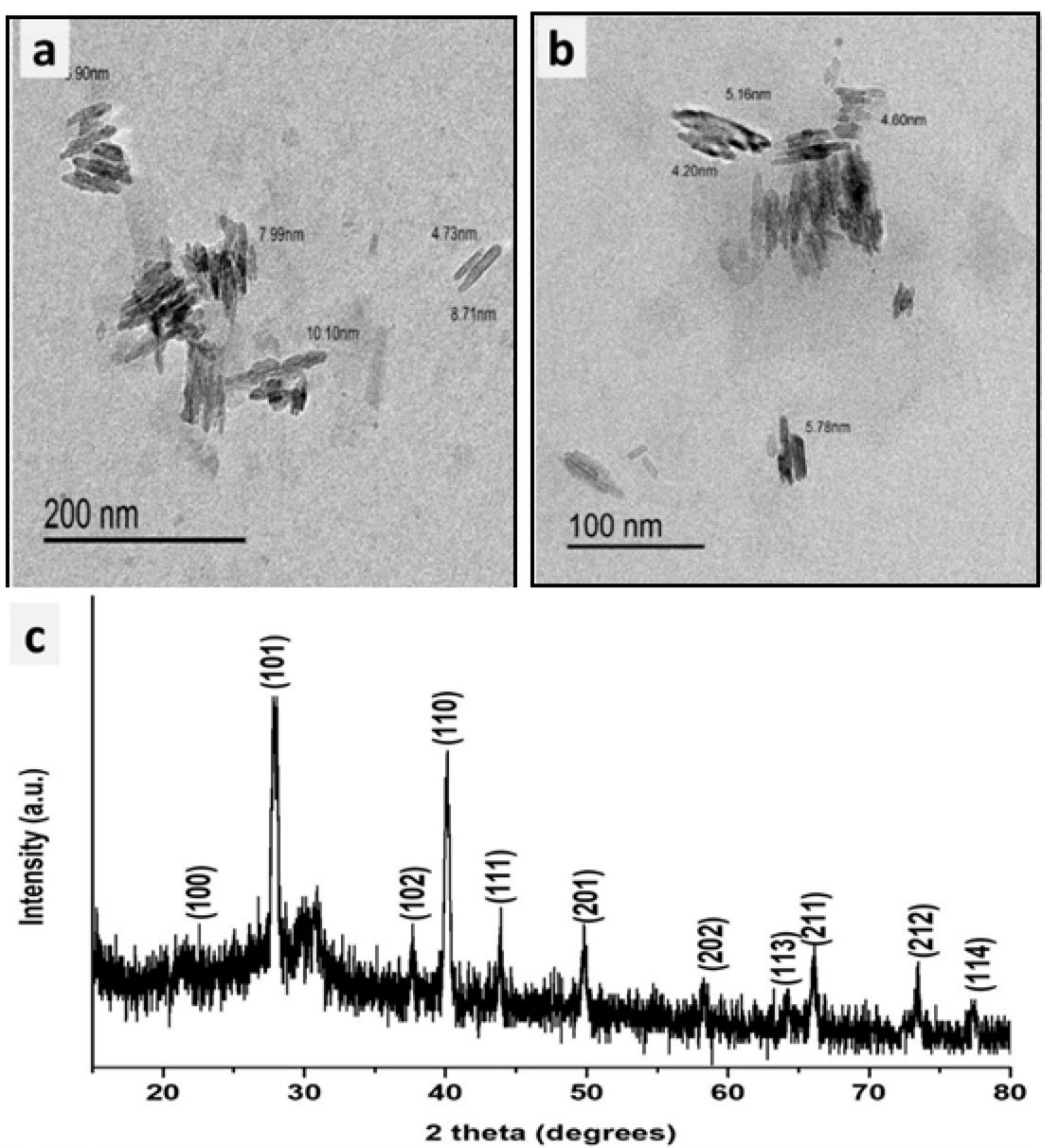
**a**,** b)** TEM micrographs (the instrument-generated scale bar is shown at the bottom left of micrographs), and **c)** XRD pattern of the prepared Te-NPs.

Transmission electron microscopy (TEM) characterization was conducted to confirm the formation of Te particles at the nanoscale and to examine their morphology. The TEM micrographs in Fig. 1 (a, b) display that the prepared TeNPs exhibit a nanorod morphology. The nanorods exhibit lengths ranging approximately from 5 to 102 nm, while their diameters were in the range of 2–11 nm, indicating successful synthesis. As shown in the TEM micrographs, the TeNPs were well dispersed within the colloidal solution, with minimal aggregation, revealing a relatively uniform and monodispersed distribution. Amorphous Te nuclei firstly form in the colloidal solution, and then deposit onto crystalline tellurium seeds, encouraging particle growth via a solid–solution–solid transformation mechanism. Crystalline tellurium tends to an anisotropic crystal structure, therefore crystal growth occurs along the < 001 > direction, which prefers the formation of rod structures and leads to the development of nanorods^27^, as shown in Fig. 1 (a, b).

To further confirm the crystalline structure of the prepared TeNPs, XRD analysis was carried out. Figure 1(c) shows several sharp and intense peaks, indicating that the prepared TeNPs appear a high degree of crystallinity. The observed diffraction peaks are corresponded to (100), (101), (102), (110), (111), (201), (202), (113), (211), (212), and (114) crystal planes. These peaks are compatible with the hexagonal phase of Te, matching well with the standard data reported in the JCPDS card No. 36-1452, confirming the successful synthesis of crystalline TeNPs^28,29^.

### Leather characterization before and after decoration with TeNPs

#### Effect of temperature, time, and pH on the antibacterial efficacy of leather treatment against pseudomonas aeruginosa

The data showed that antibacterial activity increased with temperature up to an optimal point. At room temperature, the zone of inhibition was 38 mm, rising significantly and peaking at 42 mm at 50 °C then it decreased to 36 at 70 °C. This suggests that elevated temperatures enhance the release or reactivity of TeNPs on the leather surface, potentially by increasing nanoparticle mobility and interaction with bacterial cell membranes. The thermal energy likely facilitates better dispersion of TeNPs within the leather matrix, improving their biocidal efficacy. These results confirm that the antibacterial performance of TeNPs/leather is strongly pH-dependent, with optimal activity at neutral pH (pH 7), where the zone of inhibition against *P. aeruginosa* reached 42 mm.

The antimicrobial performance of TeNPs/ leather is clearly pH dependent. The inhibition zone was 37 mm at pH 5, peaked at 42 mm at pH 7, then slightly declined to 41 mm at pH 9. The optimal activity at neutral pH suggests that TeNPs are most stable and reactive under these conditions. At acidic pH, partial aggregation or reduced surface charge may limit effectiveness, while at alkaline pH, the slight decrease may be due to surface oxidation or altered nanoparticle surface chemistry affecting their interaction with microbial targets.

**Fig. 2:**
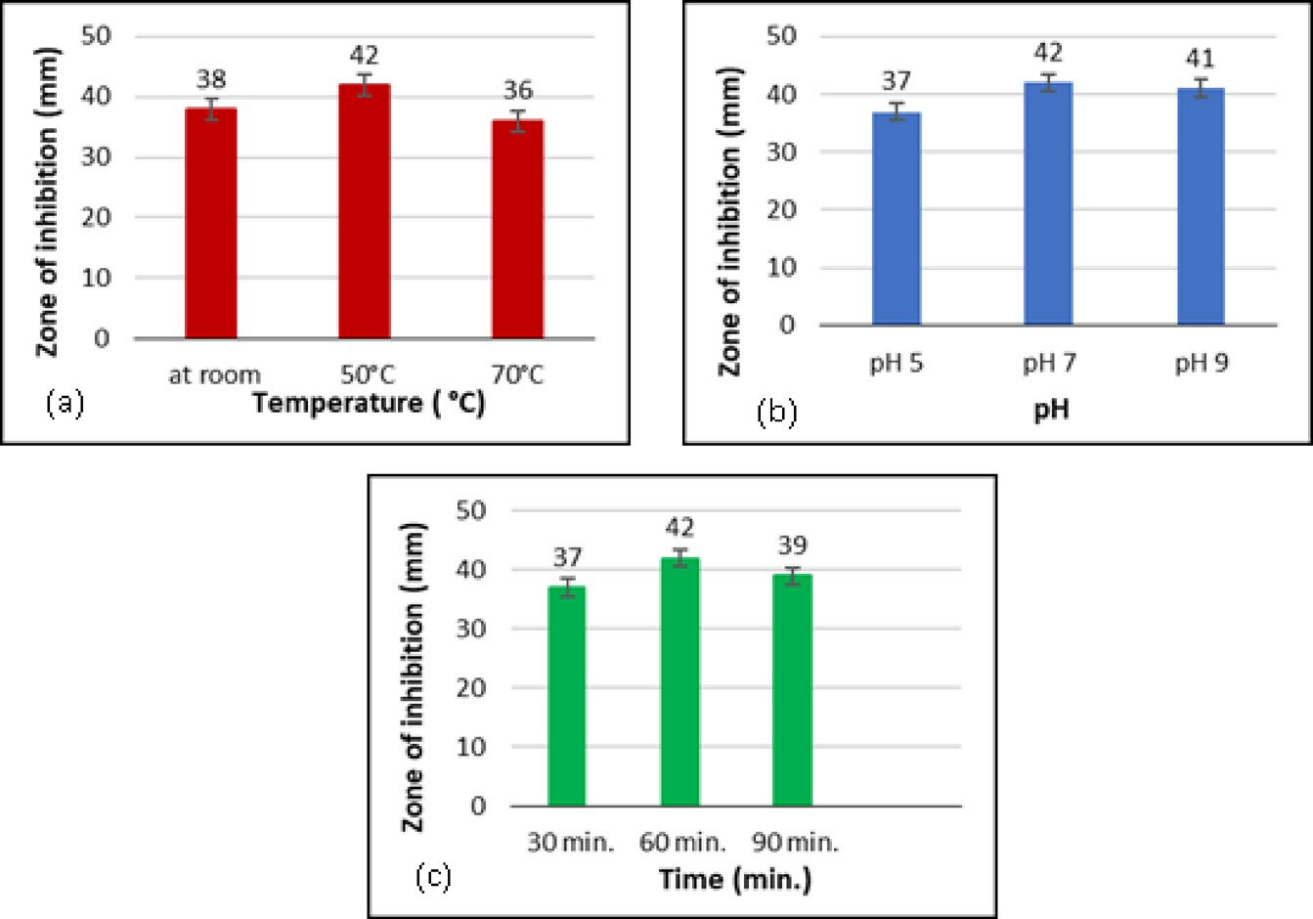
Effects of **(a)** temperature, **(b)** pH, and **(c)** treatment time on the antibacterial efficacy of TeNP-treated leather against *Pseudomonas aeruginosa*, expressed as the zone of inhibition (mm). Data represent mean ± SD (*n* = 3).

The time-dependent data demonstrated a clear positive correlation between exposure duration and antibacterial activity, up to a plateau. The zone of inhibition was 37 mm at 30 min, then it increased to a maximum of 42 mm at 60 min, before slightly declining to 39 mm at 90 min. The peak at 60 min indicated the optimal treatment time for TeNPs to penetrate and act against microorganisms in leather. The slight decrease at 90 min may be attributed to nanoparticle aggregation over prolonged exposure (see Figure 2).

#### Scanning electron microscopy analysis (SEM) and energy dispersive X-ray spectroscopy (EDX) of treated leather with TeNPs

**Fig. 3:**
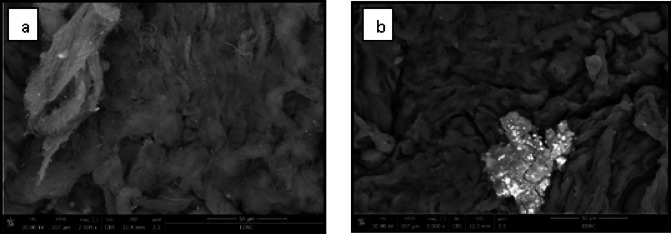
SEM Images of **(a)** untreated leather and **(b)** treated leather with TeNPs.

**Fig. 4:**
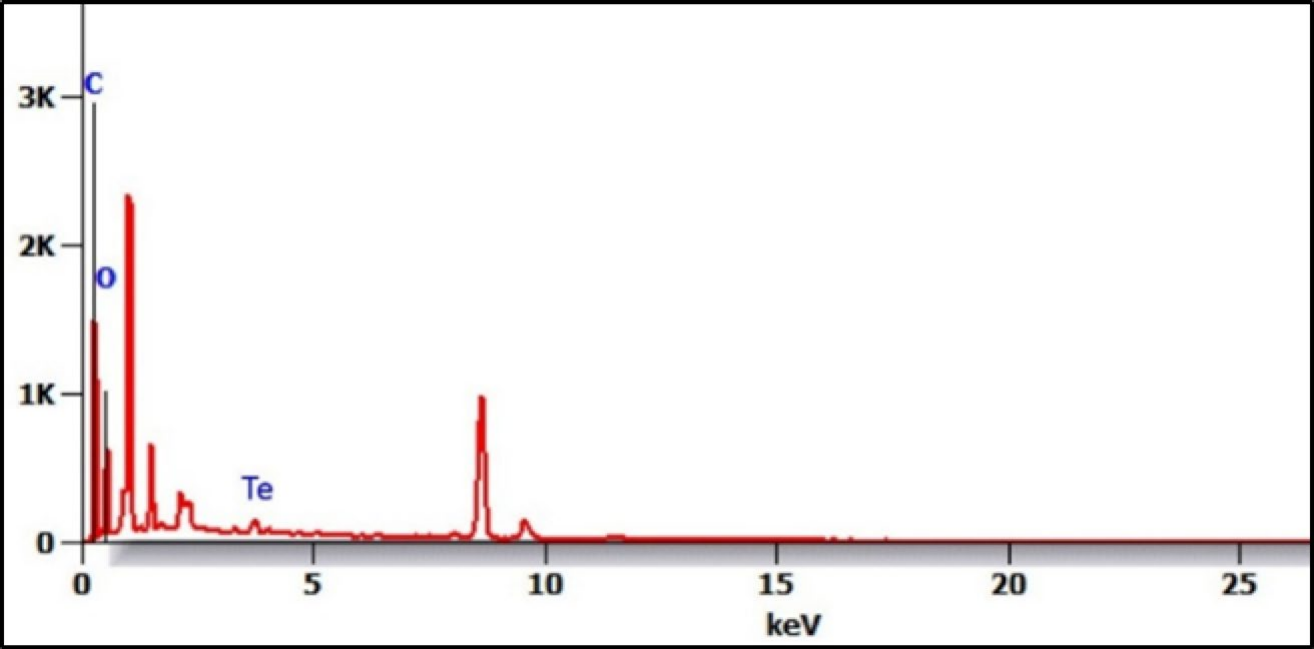
EDX Image of treated leather with TeNPs.

SEM micrographs at Fig. 3 clearly revealed dispersed tellurium nanoparticles (TeNPs) anchored on the leather surface, confirming their successful deposition. As for the EDX spectra; Te peak at ~ 3.8 keV was observed in Fig. 4, matching the characteristic Te Lα line commonly used to confirm elemental Te in nanoparticle systems^30,31^. The combination of Te peak at this energy with low Te atomic percentages in a C/O-rich matrix was closely resembles previously reported Te-nanoparticle and Te-doped carbon systems characterized by SEM/TEM-EDX, supporting the presence of Te-rich nanoscale phases on the leather surface^32,30,33^. The corresponding EDX quantitative analysis (Table 1) further confirms the incorporation of Te into the predominantly C/O leather matrix.

**Table 1:** Elemental composition of TeNPs/leather obtained from EDX analysis (wt% and at%).

		C	O	Te
*Weight%*	**Untreated leather**	37.5	47.2	-
*Atom %*		47.3	44.7	-
*Weight%*	**Treated leather with TeNPs**	48.4	46.8	4.75
*Atom %*		57.57	41.92	0.53

#### Physical properties of decorated leather with TeNPs

Table 2 showed that the mechanical properties of leather which were evaluated before and after treatment with tellurium nanoparticles (TeNPs). The untreated (blank) leather exhibited a tensile strength of 37 kgf and an elongation at break of 16%. After TeNP treatment, the tensile strength increased to 40.5 kgf, while the elongation at break decreased to 11%. These results indicate that TeNP treatment enhances the strength of leather but reduces its extensibility. The increase in tensile strength after TeNP treatment suggests that the nanoparticles interact with leather, possibly filling voids and forming additional physical or chemical bonds, which reinforce the leather structure^34^. This reinforcement makes the leather more resistant to applied loads. Conversely, the reduction in elongation at break indicates a loss of flexibility, as the fabric becomes stiffer and less capable of deforming before failure^35,36^. The increase in tensile strength and reduction in elongation support the hypothesis that TeNPs interact with the collagen network, filling existing voids and creating additional physical or chemical bonds, thereby reinforcing the leather matrix and making it more resistant to applied loads while slightly decreasing its flexibility^37^.

**Table 2:** Physical properties of untreated leather and TeNPs/leather.

Sample	Tensile strength (Kgf)	Elongation (%)
**Blank**	37	16
**Treated**	40.5	11

#### Antibacterial and antifungal properties

**Fig. 5:**
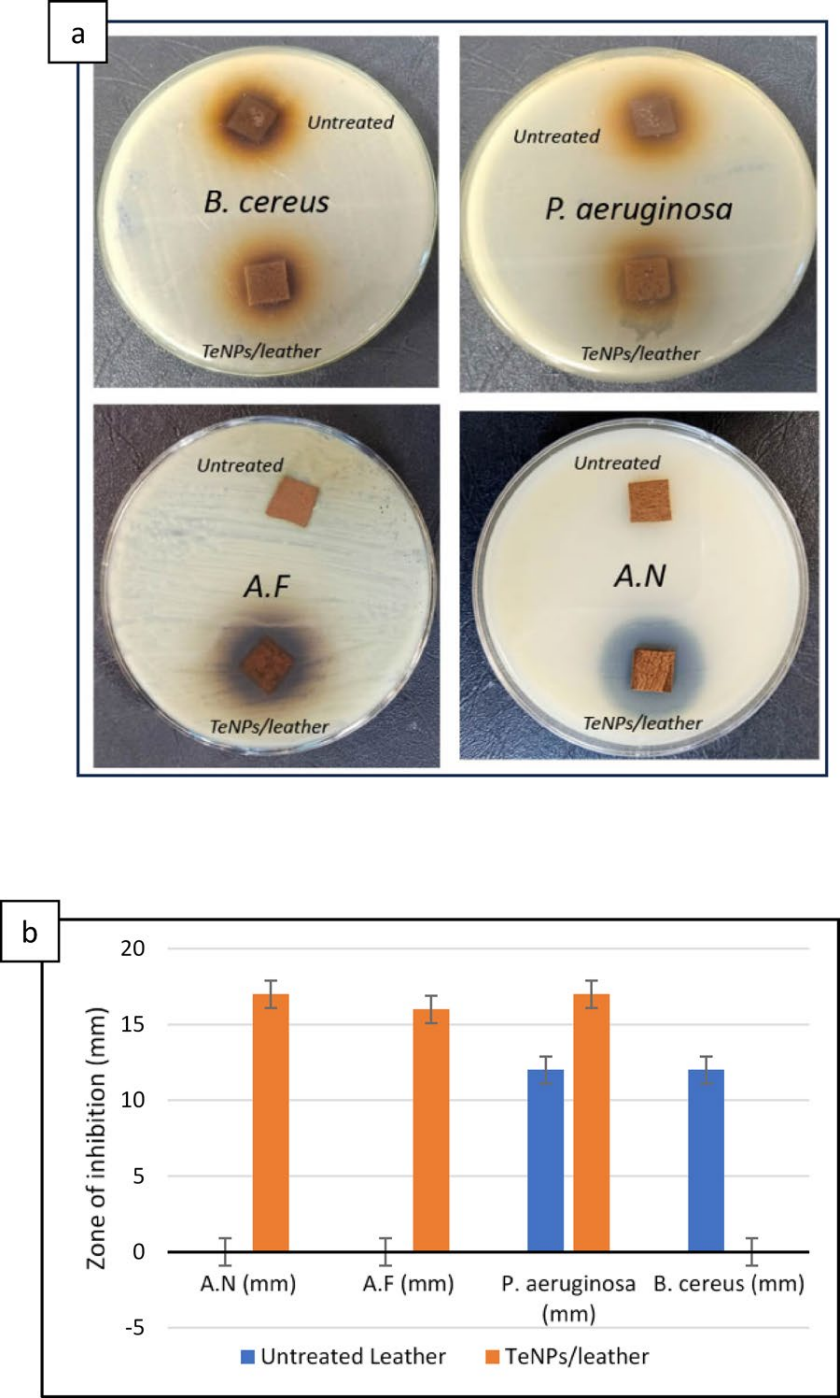
**a**,** b)** Antimicrobial activity of untreated and TeNPs-treated leather. **(a)** Agar well diffusion assay showing the zones of inhibition against *B. cereus*, *P. aeruginosa*, *A. flavus* (A.F), and *A. niger* (A.N). **(b)** Comparative bar chart of the inhibition zone diameters (mm) for untreated leather and TeNPs/leather.

The antimicrobial performance of TeNP-treated and untreated leather is presented in Fig. 5; Table 3. Untreated leather showed no antifungal activity against *A. niger* or *(A) flavus* (0 mm ZOI) and moderate antibacterial activity against both *P. aeruginosa* and *(B) cereus* (12 mm each), attributable to residual tanning chemicals selectively active against Gram-positive organisms^38^. TeNP treatment markedly enhanced antifungal activity (17 and 16 mm against *A. niger* and *(A) flavus*, respectively) and antibacterial activity against *P. aeruginosa* (17 mm), while inhibition of *(B) cereus* was eliminated (0 mm), consistent with the known preferential potency of TeNPs toward Gram-negative bacteria^39^ and a possible reduction in bioavailability of residual tanning agents following TeNP incorporation into the collagen matrix. These results compare favorably with Ag NP- (14–18 mm^8^, SeNP- (13–16 mm^11^, and TiO₂-based leather finishes (10–15 mm^9^, with the additional advantage of consistent antifungal efficacy that silver-based systems frequently lack.

The superior performance of TeNPs is attributable to their multi-target mechanism, which is fundamentally distinct from Ag NP ion-release-dependent action and encompasses: (1) ROS generation via Te²⁻/Te⁰/Te⁴⁺ redox cycling; (2) covalent binding to thiol groups of membrane proteins and respiratory enzymes including thioredoxin reductase; (3) direct nanoscale membrane disruption; and (4) intracellular accumulation of toxic tellurium intermediates^22,39^. This multi-target mode of action substantially reduces the risk of resistance development. From a production standpoint, TeNP synthesis using low-cost plant biomass and potassium tellurite under ambient aqueous conditions avoids the high precursor costs of noble metal-based systems, and the simple finishing process is fully compatible with existing tanning operations without capital investment; extension to chrome-free leather substrates is designated as a priority for future work to further enhance sustainability. All tests were performed in triplicate and inhibition zones are reported as mean ± SD (Table 3).

**Table 3:** Zone of inhibition (mm) of untreated leather and TeNPs/leather (mean ± SD, *n* = 3).

Sample Type	A.*N* (mm)	A.F (mm)	*P*. aeruginosa (mm)	B. cereus (mm)
**Untreated Leather**	0	0	12	12
**TeNPs/leather**	17	16	17	0

## Conclusion

In this study, leather was successfully functionalized with green-synthesized tellurium nanoparticles (TeNPs), as verified by TEM and XRD analyses of crystalline TeNPs together with SEM/EDX evidence confirming their anchoring on the leather surface. TeNP-treated leather exhibited pronounced antibacterial and antifungal activity, with substantial inhibition of representative bacterial and fungal strains such as *Pseudomonas aeruginosa*, *Aspergillus niger*, and *Aspergillus flavus* compared to untreated leather. Concurrently, tensile strength increased while elongation remained within an acceptable range, indicating that TeNP incorporation reinforced the leather matrix without compromising its essential mechanical performance. Collectively, these results highlight TeNP-functionalized leather as a promising hygienic, high-value material for advanced footwear and related applications requiring robust control of microbial contamination. The present work focused on initial structural, mechanical, and antimicrobial evaluation; long-term durability under repeated mechanical stress, washing, or environmental aging was not assessed and remains an important topic for future investigation. Because TeNP treatment can be integrated after conventional tanning using simple aqueous baths and moderate temperatures, it is compatible with existing industrial infrastructure and can be implemented without major capital investment. The simplicity and low cost of the TeNP finishing process, combined with enhanced antimicrobial protection and tensile strength, position this material as a strong candidate for premium footwear, automotive, and medical leather markets. Before any commercial application, however, cytotoxicity evaluation against human dermal fibroblast and HaCaT keratinocyte cell lines via MTT assay is required, given the intended skin-contact use. Long-term durability and aging behavior of TeNP-treated leather will also be addressed in future investigations.

## Supplementary Information

Below is the link to the electronic supplementary material.

Supplementary Material 1

## Data Availability

The datasets presented during or analyzed during the current study are available from the corresponding author upon reasonable request.
