# Supplementary material for: Green-synthesized tellurium nanoparticles as a multifunctional leather finishing agent: antimicrobial and mechanical enhancement
Source: Sci Rep. 2026 Jun 16;16:18712. doi: 10.1038/s41598-026-57078-0 (PMC13273169; doi:10.1038/s41598-026-57078-0)
Supplement: Supplementary file 1 — Supplementary Material 1 [file 41598_2026_57078_MOESM1_ESM.pdf]

Table S1. Pre-tanning and tanning steps applied in the experiment

Step	%	Description Added	Time (min)	Notes
**Pre-soaking**	200	Water	15	• Drain
**Second soaking**	200	Water	15	• Drain
	0.25	Soap		
**Main soaking**	200	Water	15	• Percentages were calculated based on soaking weight.<br>• Drum speed 2cyc./min.<br>• Overnight then drain.<br>• pH (9:9.5).
	0.25	Soap		
	0.5	Soda ash		
**Unhairing**	100	Water	120	• Running was intermittent (run 20 min and stop 20 min)..<br>• Drum speed 3cyc./min.<br>• Overnight with 5 min running every hour for 16 h.<br>• pH(12.5:13).
	0.25	Soap		
	3	Lime		
	1.5	Sodium sulphide		
**Deliming**	20	Water	30	• Percentages were calculated based on soaking weight.<br>• Drum speed 5cyc./min .
	1	Ammonium sulphate		
	0.5	Soap	30	
	1.5	Ammonium chloride		
**Degreasing**	0.75	Degreasing Agent	30	
	0.5	Soap		
**Bating**	0.05	Orpone bate	30	• Orpone concentration 7000 IU.<br>• Bating ph(8.5:9).
**Washing**	200	Water	15	• Drain and Wash (4).
**Pickling**	100	Water	15	• Hides were drummed with water and salt for about 15 min then acids added gradually.<br>• Bé = 9 – (ph 3:3.5).<br>• Formic acid dilutes a rate of 1:5 while sulfuric acid 1:10.<br>• Overnight with 1 min/hour.
	10	Salt		
	0.25	Soap	40	
	0.6	formic acid		
	1.5	sulfuric acid	180	
**Tanning**	100	Water	10	• Bé = 7<br>• Overnight with 1 min/hour.<br>• pH = 4<br>• Check boiling test before next step.<br>• Drum speed 8 cyc./min.
	10	Salt		
	10	Mimosa	60	
	2	Format sodium	480	
**Hides horsed up for three weeks then shaving and weight leathers**				

**Naturalization**	100	Water	60	• pH = 4.5-5<br>• Drain and wash 2
	0.25	Soap		
	0.8	Format sodium		
	0. 1	Sodium bicarbonate		
**Re-tanning**	2	Acrylic	30	
	1	Phenolic syntan	60	
	5	Mimosa		
	2	Quebracho		
**Fatliquoring**	4	synthetic sulphited oil	60	• Water temperature 40 C.<br>• Check fatliqour in float before next step.
	1	Sulphonated oil		
**Fixation**	1	Formic acid	30	• Drain and wash.<br>• pH = 3.5<br>• Hourse up and overnight.<br>• Next day samming and drying
	1	Formic acid	30	
